# STROBE-Long-Term Exposure to Ambient Fine Particulate Air Pollution and Hospitalization Due to Peptic Ulcers

**DOI:** 10.1097/MD.0000000000003543

**Published:** 2016-05-06

**Authors:** Chit-Ming Wong, Hilda Tsang, Hak-Kan Lai, Thuan-Quoc Thach, G. Neil Thomas, King-Pan Chan, Siu-Yin Lee, Jon G. Ayres, Tai-Hing Lam, Wai K. Leung

**Affiliations:** From the School of Public Health, Li Ka Shing Faculty of Medicine, The University of Hong Kong, Faculty of Medicine Building, Pokfulam (CMW, HT, HKL, TQT, KPC, THL); Institute of Applied Health Research, The University of Birmingham, Edgbaston, Birmingham, UK (GNT, JGA); Department of Health, Wu Chung House, Wan Chai (SYL); and Department of Medicine, Li Ka Shing Faculty of Medicine, The University of Hong Kong, Queen Mary Hospital, Pokfulam Road, Hong Kong (WKL).

## Abstract

Little is known about the effect of air pollution on the gastrointestinal (GI) system. We investigated the association between long-term exposures to outdoor fine particles (PM_2.5_) and hospitalization for peptic ulcer diseases (PUDs) in a large cohort of Hong Kong Chinese elderly.

A total of 66,820 subjects aged ≥65 years who were enrolled in all 18 Government Elderly Health Service centers of Hong Kong participated in the study voluntarily between 1998 and 2001. They were prospectively followed up for more than 10 years. Annual mean exposures to PM_2.5_ at residence of individuals were estimated by satellite data through linkage with address details including floor level. All hospital admission records of the subjects up to December 31, 2010 were retrieved from the central database of Hospital Authority. We used Cox regression to estimate the hazard ratio (HR) for PUD hospitalization associated with PM_2.5_ exposure after adjustment for individual and ecological covariates.

A total of 60,273 subjects had completed baseline information including medical, socio-demographic, lifestyle, and anthropometric data at recruitment. During the follow-up period, 1991 (3.3%) subjects had been hospitalized for PUD. The adjusted HR for PUD hospitalization per 10 μg/m^3^ of PM_2.5_ was 1.18 (95% confidence interval: 1.02–1.36, *P* = 0.02). Further analysis showed that the associations with PM_2.5_ were significant for gastric ulcers (HR 1.29; 1.09–1.53, *P* = 0.003) but not for duodenal ulcers (HR 0.98; 0.78 to 1.22, *P* = 0.81).

Long-term exposures to PM_2.5_ were associated with PUD hospitalization in elder population. The mechanism underlying the PM_2.5_ in the development of gastric ulcers warrants further research.

## Abstract 2

Supplemental Digital Content is available in the text

## INTRODUCTION

Air pollution is a major public health hazard, particularly in developing countries.^1^ The World Health Organization estimated substantial premature death attributable to ambient air pollution in 2012.^2^ In particular, there is a causal association between air pollution and morbidity or mortality from cardiorespiratory diseases.^3^ Among various World Health Organization criterion pollutants, particulate matter (PM) which is emitted from burning of fossil fuels in vehicles, shipping and power generators, is often considered the most relevant to public health intervention.^4^ PM is classified by its aerodynamic diameter of less than 2.5 μm into PM_2.5_ (particles less than 2.5 μm in aerodynamic diameter), which can enter the blood stream via the alveolar capillaries of the lungs with a higher risk to cause serious health problems.^5^ By using chemistry model to estimate exposure based on emission source categories, air pollution, mostly PM_2.5_, in urban and rural environments, would lead to 3.3 million premature deaths per year worldwide, predominantly in Asia.^6^

Recent long-term studies have estimated the horizontal spatial variation of PM using geospatial or dispersion modeling and satellite information as proxy indicators of exposure in individual residential areas.^7–10^ However, measurement errors in estimating the exposure exist if the spatial variation in the vertical dimension has not been taken into account. Considering the vertical dimension is particularly important for studies in populations mainly living in high-rise buildings like Hong Kong. People who stay near the ground level could be exposed to as much as 5-time higher pollutant concentrations than those staying on floors above ground level, depending on the street canyon characteristics.^11^

Peptic ulcer disease (PUD) is a very common gastrointestinal (GI) disease in Hong Kong. It is estimated that more than 18.6% of all hospitalizations for GI diseases were due to PUD in Hong Kong in 2011.^12^ Although the majority of PUD is caused by chronic *Helicobacter pylori* (*H pylori*) infection and the use of nonsteroidal inflammatory drugs (NSAIDs) or aspirin, the “*H pylori*-negative ulcers” in non-NSAID users appear to be on the rise, particularly in Asia.^13^ Factors other than *H pylori* and NSAIDs may play an important but yet undefined role on the pathogenesis of this group of PUD.^14^

In the present study, we studied the effects of long-term exposure to PM_2·5_ on hospital admissions for PUD in a large group of elderly persons from all 18 districts of Hong Kong. These subjects had participated in the Government Elderly Health Service during the recruitment period and were followed up for 10 to 13 years. The 1st hazard admissions to hospital due to PUD during the follow-up period were regressed on the PM_2.5_ concentration estimated at their residential addresses, taking into account both the horizontal and vertical spatial variations.

## METHODS

### Subjects

From July 1998 to December 2001, 66,820 adults aged 65 years or above were enrolled by the Elderly Health Service of the Department of Health of the Hong Kong Government for participation in a client-oriented primary health care service that aimed to promote the health of elderly population and to enhance self-care ability so as to minimize illness and disability (www.info.gov.hk/elderly). These Elderly Health Centers were located one in each of all the 18 districts in Hong Kong, which provide clinical services of health assessment, physical check-up, counseling, curative treatment, and health education to the elderly clients. All participants had their socio-demographic data, lifestyles, health, and anthropometric measurements recorded at baseline.^10^ During the recruitment, subjects agreed that the data they provided could be used in research to improve the health of elderly population. The database was stored and managed by the Department of Health, and the anonymous data were available for public health research. Pseudo-identifiers were used to match up records from the Elderly Health Centre to the Hospital Authority data. The investigators and the research workers of the project were blinded to the link between the identity and personal data of the individual subjects. The study protocol was approved by the Institutional Review Board of The University of Hong Kong/Hospital Authority Hong Kong West Cluster and the Ethics Committee of Department of Health of Hong Kong Government.

### Exposure Model to Air Pollution and Estimation of Residential Exposure

The annual mean concentrations (in μg/m^3^) of PM_2.5_ from 1998 to 2011 were measured hourly in 5 urban locations by tapered element oscillating microbalance of the Environmental Protection Department of the Hong Kong Government (http://epic.epd.gov.hk/EPICDI/air/station/). All the selected air pollutant monitoring stations (n = 5) were located throughout the metropolitan areas with height ranging from 11 to 27 m above the ground. In all the monitoring locations, we determined vertical height (m) above the mean sea level and the satellite-derived surface extinction coefficients (SEC) (within the 1 × 1 km grid area of the station).^15^ We then fitted regression models to estimate annual PM concentrations using SEC and inverse vertical height as independent variables and validated the approach by comparing with data estimated from leaving out 1 station and by comparing with subset of the data estimated from an independent study (eAppendix).

We matched all residential addresses of the subjects with the SEC data by geocoding and estimated the vertical height of the residence based on the floor level on which they live. Using the exposure model, we determined the annual mean exposures to PM_2.5_ for each residential location.

### Hospital Admissions for PUD

Hospitalization records of all participants from 1998 to 2010 were retrieved from the electronic health record system of the Hospital Authority of Hong Kong, which manages all public hospitals in Hong Kong, that accounts for 88% of all hospital beds and 78% of total hospital admissions locally.^16^ We captured the primary cause of hospitalization using the International Statistical Classification of Diseases (ICD9-CM) codes for specific site of PUD (531–532) and its subcategories: gastric ulcer (531) and duodenal ulcer (532) during the study period of all enrolled subjects.^17^ We also included in the analysis another GI disease: reflux esophagitis (530.1×, 530.2, 530.81, 530.82, and 530.85). To assess the specificity of effects, we assessed the causes of hospital admissions which were considered to be unrelated to PM exposure including poisoning and injuries (800–999), diseases of skin and subcutaneous (680–709), and oral structures (520–529). To assess the sensitivity of our spatial exposure model of PM in this cohort, hospital admissions due to cardiovascular and respiratory diseases (390–459, 460–519), which are known to be associated with PM exposure, were included.

### Individual, Ecological, and Environmental Covariates

We determined the impact of individual, ecological, and environmental covariables as potential confounding factors for hospital admissions. Individual covariates were age, gender, body mass index, and the presence of medical illnesses that require regular medical care (including hypertension, heart diseases, diabetes, chronic obstructive pulmonary disease [COPD], stroke, and other diseases), smoking status (never, quit, and current smoker), exercise frequency (days per week), education level (secondary or above, primary, and below primary), and personal monthly expenditure (US$ < 128, 128–384, and ≥385) that were available in the questionnaire (eAppendix Table 1).^18^ Based on the Census Statistics of Hong Kong in 2001, we determined ecological covariates including percentage (%) of elderly subjects (aged 65 or elderly), % of tertiary education, and average monthly domestic household income in the 197 small areas, namely Tertiary Planning Units of Hong Kong. Environmental covariates included % of smokers in 18 districts of Hong Kong as proxy for exposure to environmental tobacco smoke in each year.^19,20^

### Statistical Analysis

We used Cox proportional hazards model to estimate the hazard ratio (HR) for every 10 μg/m^3^ increase of long-term exposure to PM_2.5_ concentration on hospital admissions related to 1st diagnosis of PUD after the baseline year with adjustments for individual, ecological, environmental covariates, and the year-to-year changes in exposure to take account of potential changes from the baseline year. We put into the model, the baseline year PM_2.5_ as time-independent variable and the year-to-year change which was the difference between the annual average at the year of hospitalization and at baseline year, as the time-dependent variable. We used time-on-study as a timescale and the estimated exposure in the baseline year to represent long-term exposure.

### Sensitivity Analysis

To reduce the possibility that the association between PUD and PM_2.5_ was confounded by comorbidities of the subjects, we performed 4 different sensitivity analyses. In the 1st model (covariate adjustment I), we adjusted with an indicator variable for previous hospitalizations due to ischemic heart disease (ICD-9 410–414), stroke (430–438), COPD (490–496), or type 2 diabetes mellitus (250.0, 250.2). These diseases have been previously shown to be associated with air pollution or the use of antiplatelet therapy that increases the risk of PUD.^21–24^ For the 2nd model (covariate adjustment II), we adjusted in the main model another indicator variable for the presence of cardiopulmonary diseases and diabetes as reported by subjects at baseline, while not including other self-reported diseases. For the 3rd model (stratification I), we removed all subjects from the analyses with previous hospitalizations due to the 4 preexisting diseases described in the 1st model. For the 4th model (stratification II), we removed all subjects from the analyses with history of hospitalizations due to PUD during the baseline year. Cox models were performed using the command PHREG in SAS 9.2 (SAS Institute, Inc., Cary, NC). All authors had access to the study data and had reviewed and approved the final manuscript.

## RESULTS

### Concentrations of PM_2.5_

**FIGURE 1:**
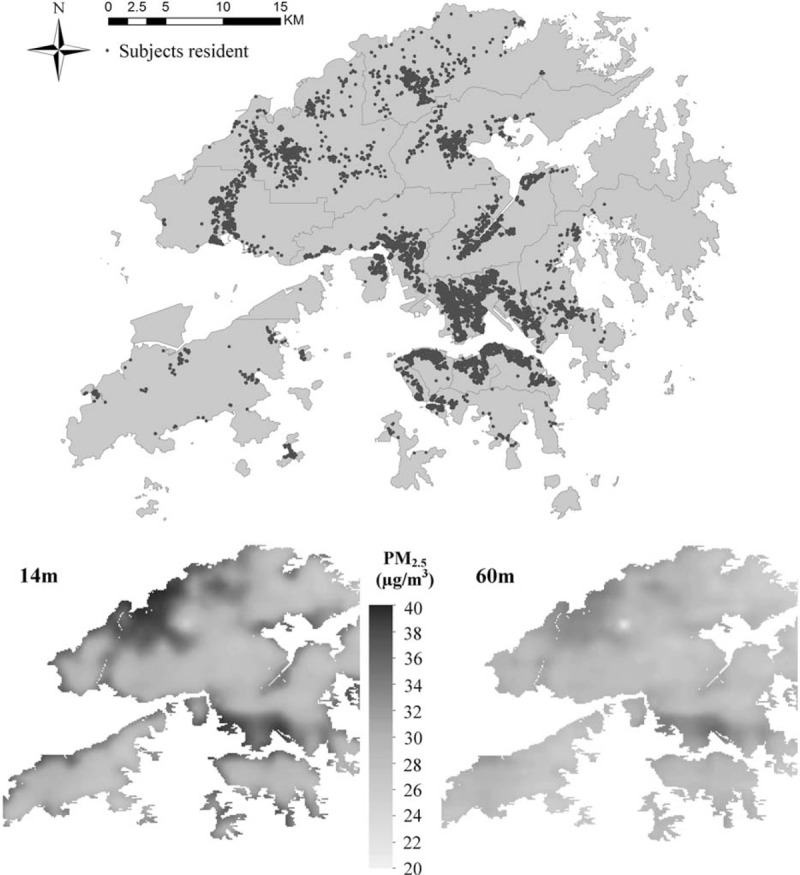
Subject's residential location and the estimated particles less than 2.5 micrometers in aerodynamic diameter (PM_2.5_) annual mean concentrations at lower (14 m) and higher (60 m) heights above ground level at baseline. Subject's residential location and the estimated annual mean PM_2.5_ concentrations at different heights above ground level at baseline. The lower (14 m) and higher (60 m) heights represent the 25th and 75th percentiles of height above ground level for the cohort's residential locations. Height from mean sea level to the ground level was taken into account in the exposure model.

The overall mean concentrations of PM_2.5_ recorded by the Environmental Protection Department monitors from 1998 to 2010 were 37.7 μg/m^3^ (standard derivation [SD] 7.1 μg/m^3^), and the annual mean concentrations (95% central range) estimated at individual residential locations in the baseline year was 33.7 μg/m^3^ (29.5–39.8 μg/m^3^) (Figure 1). The patterns of PM_2.5_ concentrations were similar among the 5 monitoring stations during the study period (eAppendix Figure 1).

### Hospitalizations for PUD

A total of 66,820 subjects were included in the initial study cohort. Among them, completed geocoding and satellite information was available in 60,273 subjects (90.1%; missing data due to missing individual-level covariates: 0.2%, problems in geo-coding: 8.1%, or problems in satellite data: 1.5%) who were included in the Cox model. The average age of these subjects was 72.1 years. There were 66% female and 71% never smokers (Table 1). The mean follow-up in the study was 10.1 (SD 2.9) years and the total follow-up was 664,927 person-years.

**TABLE 1:**
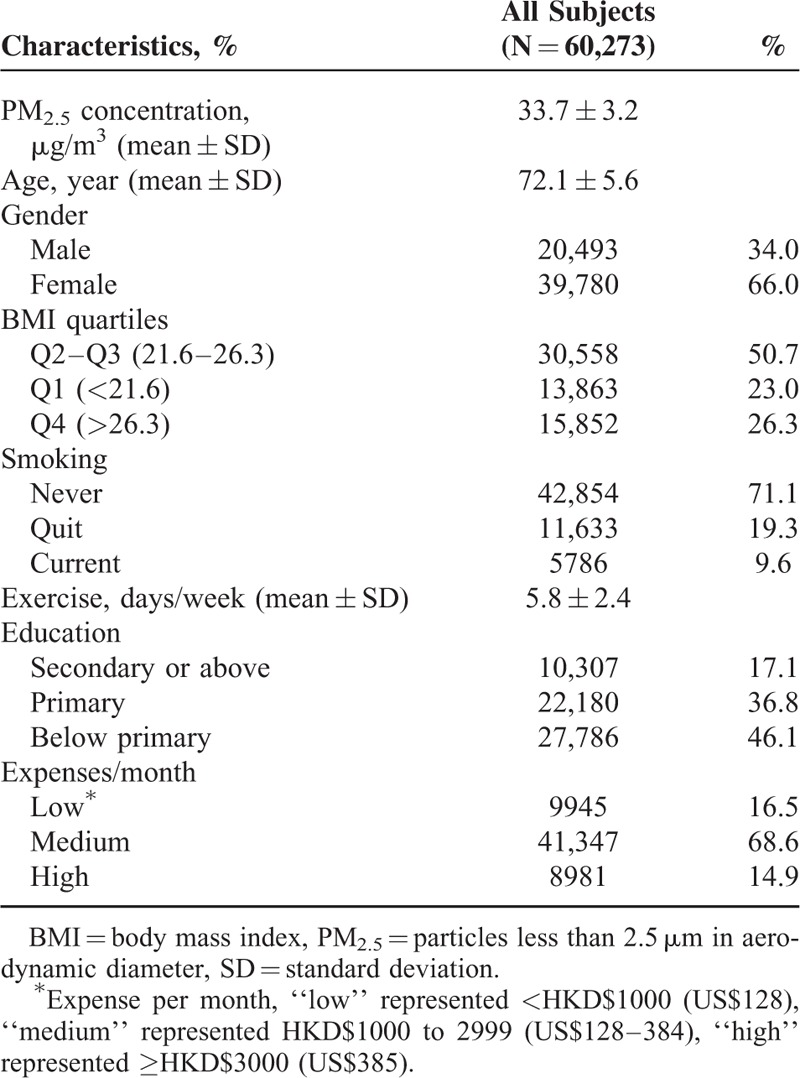
Characteristics of Subjects in Baseline (1998–2001)

During the follow-up period, 1991 (3.3%) subjects had been hospitalized for the diagnosis of PUD, with a mean length of stay of 7.9 (SD 12.3) days. The factors which were significantly associated with PUD hospitalizations included PM_2.5_, elderly age, male gender, body mass index in 4th quartile (>26.3 kg/m^2^), ever smoked (current or former smokers), low education level (primary or below), coexisting medical diseases that required regular medical care, and living in a community with low education level and with more smokers (Table 2).

**TABLE 2:**
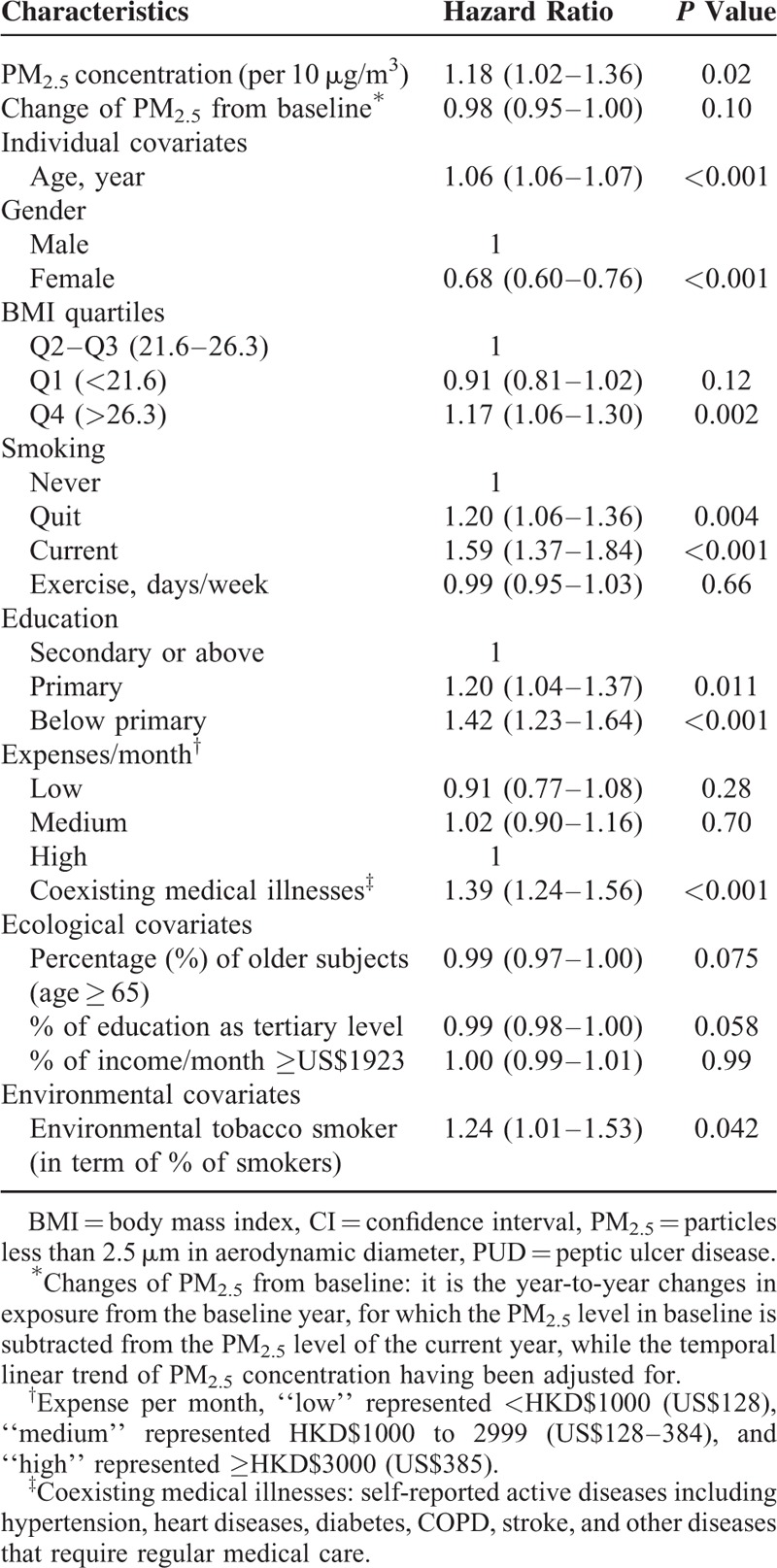
Hazard Ratios (95% CI) of Individual, Ecological, and Environmental Covariates on Hospital Admission for PUD

After adjusting for potential confounding variables, the HR (95% confidence interval) estimate for hospitalization per 10 μg/m^3^ increase of PM_2.5_ was 1.18 (1.02–1.36) for PUD (n = 1991), 1.29 (1.09–1.53) for gastric ulcer (n = 1175), and 0.98 (0.78–1.22) for duodenal ulcer (n = 816) (Table 3). There was however no significant association between peptic ulcer bleeding and PM_2.5_ levels (HR 1.01; 0.81–1.27). For all other control health outcomes considered, the estimates were not statistically different from unity, while there was a significant association between PM_2.5_ exposure and cardiovascular and respiratory diseases (Table 3).

**TABLE 3:**
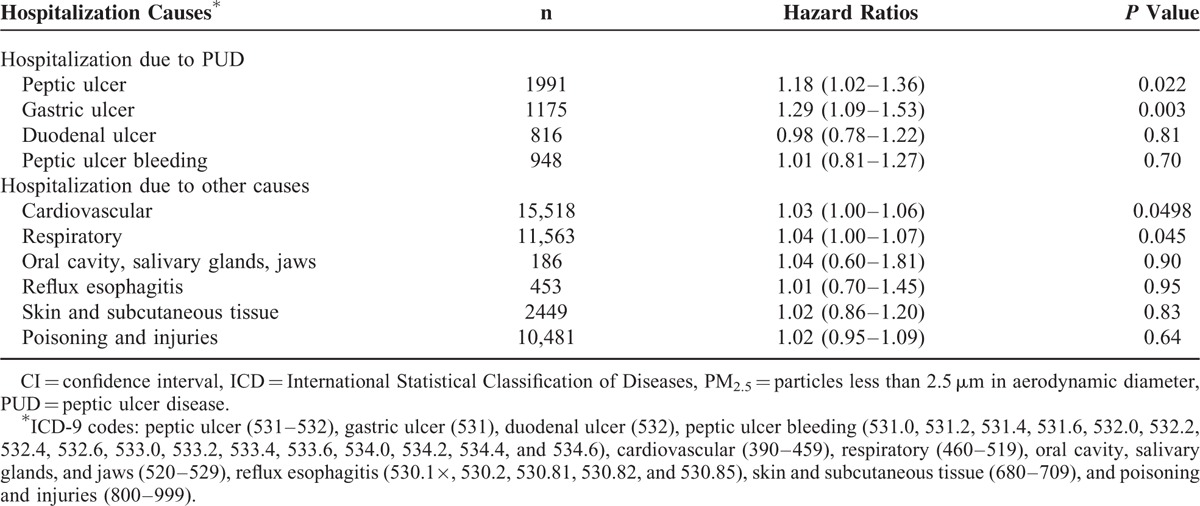
Hazard Ratios (95% CI) of Hospitalization Due to PUD or Other Causes Per 10 μg/m^3^ of PM_2.5_ (N = 60,273)

### Sensitivity Analyses

The association between PM_2.5_ and PUD remained significant after controlling for preexisting medical diseases by 2 different covariate adjustments. The magnitude of HR for PUD was similar to the original values after adjusting for coexisting medical diseases or after removing subjects with comorbidities or hospitalized during the baseline year (Table 4). In particular, the HR remained around 1.28 to 1.29 for gastric ulcer.

**TABLE 4:**
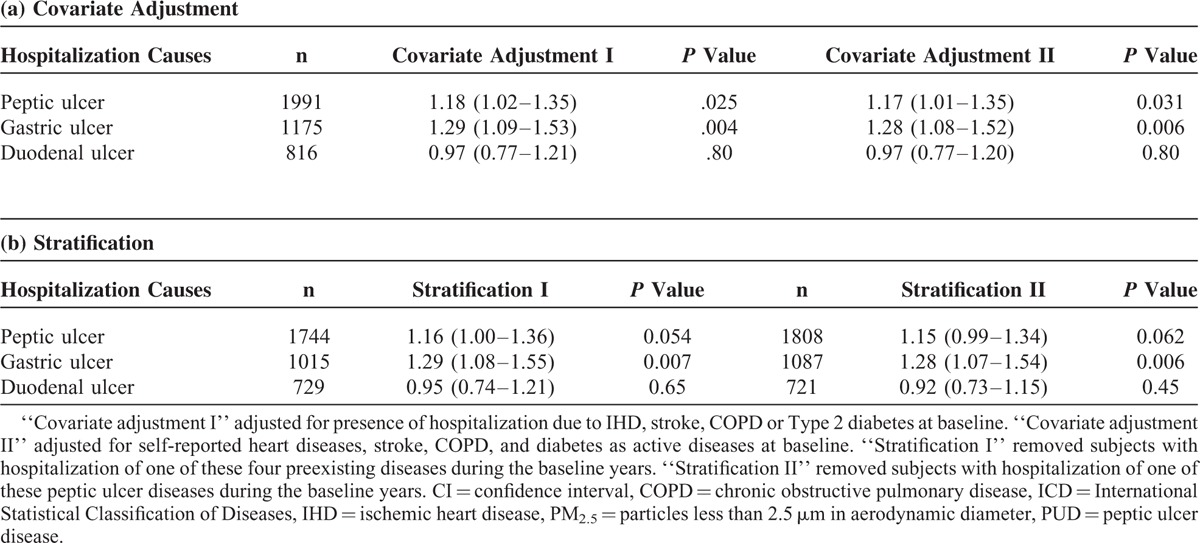
Hazard Ratios (95% CI) of PUD Hospitalization per 10 μg/m^3^ of PM_2.5_ After Controlling for Preexisting Comorbidities

## DISCUSSION

Ambient PM_2.5_ exposure has been found to be one of the leading causes of death and disability in the world.^25^ We have demonstrated for the 1st time an association between long-term exposure to PM_2.5_ and PUD among elderly people in Hong Kong. Specifically, a significant association for gastric ulcer has been shown both before and after adjustment for other potential confounding factors including baseline medical conditions and hospitalization due to other diseases. Plots of the monthly counts of hospitalization for PUD along with PM_2.5_ and particles less than 10 micrometers in aerodynamic diameter (PM_10_) monthly concentration from 2001 to 2011 (eAppendix Figure 2) suggested that there were seasonal variations showing winter (December–February) peaks. The patterns were congruent to the global patterns of PUD seasonality and lent support to the hypothesis of this study that PUD hospitalizations are related to environmental factors.^26^ However, the covariations between PUD and PM_2.5_ concentration should be studied with adjustment for confounding.

In this study, we used a reliable and comprehensive prospective health database from the Department of Health, which include a large cohort of 66,820 elderly people who participated in the study and were being followed up for more than 10 years. The baseline medical and socio-economic information of the participants were collected and their subsequent hospital admission records were retrieved from the central database of the Hospital Authority, which captures all hospital admission records of public hospitals in Hong Kong.^27^ We believe that the data are very comprehensive and would be representative of local situations in Hong Kong as the majority of elderly patients are admitted to public hospitals through the accident and emergency department when needed. With the aging population and the air pollution in Hong Kong, the potential impact on health care utilization should not be underestimated.

PM_2.5_ typically originates from all types of organic material combustion in motor vehicles, power plants, and industrial processes in Hong Kong. PM_10_ (comprising about 70% PM_2.5_) mainly contains chemical compounds (nitrate [27.9%], sulfate [19.5%], and ammonium [8.6%]), as well as carbonaceous (OC [15.9%] and EC [7.6%]) and metal species (Na^+^, Cl, Ca, K^+^, Fe, Mg, Al, Pb, Mn, V, As, Ni, and Cd) (eAppendix Table 2). The chronic stimulation from long-term PM_2.5_ exposure can induce systemic inflammatory responses and oxidative stress, which is triggered by acidity, transition metals or the ultrafine fraction in particles.^5^ Due to the relatively small size, PM_2.5_ can escape phagocytosis.^28^ PM_2.5_ has been linked to inflammation in blood vessels, which increases the risk of plaque deposition and atherosclerosis.^29^ However, the mechanistic pathway linking PM_2.5_ and peptic ulcer development remains elusive. Yet particles, which block sunlight and prevent absorption of vitamin D useful in enhancing intestinal calcium absorption and mobilizing osteoclastic activities, would lead to development of peptic diseases.^30^ Although air pollutants affect the respiratory or circulation system directly, the potential effects on the GI trait is biological plausible. First, human experimental studies have shown that air pollutants can enter the aerodigestive tract through the swallowing of inhaled PMs cleared from the lungs into the intestine,^31^ as well as through ingestion of contaminated food and water.^32,33^ Second, by means of a combination of factors including increased gut permeability,^34^ decreased colonic motility and clearance,^31,35^ altered gut microbial composition and metabolic function,^29,36^ air pollutants including PM can induce adverse effects in the GI tracts.^37^ In epidemiology studies, long-term exposure to higher concentrations of air pollutants including PM has been associated with an increased risk of early-onset Crohn disease,^32^ increased risk of hospitalizations for inflammatory bowel diseases,^38,39^ and increased standardized mortality ratio for peptic ulcer.^40^ Recently a case cross-over analysis showed that PM_2.5_ was inversely associated with upper GI bleeding secondary to PUD.^41^ However, the significant estimates were not replicated in another data of the study and the assessment was for short-term instead of for long-term exposure.

Apart from *H pylori*, aspirin and NSAIDs, stress is another important cause of PUD, particularly in critically ill patients.^42^ Recent studies from Japan demonstrated that both physical and psychological stresses related to earthquakes play a pivotal role on the pathogenesis of PUD.^43^ We hypothesize that the association between PM_2.5_ exposure and PUD, particularly for gastric ulcer, is also mediated through stress and inflammation. It is interesting to note that only gastric ulcer appears to be associated with the long-term PM_2.5_ exposure in the present study. Although duodenal ulcer is principally considered to be an acid related disease, the pathogenesis of gastric ulcer is likely related to breakdown of mucosal defense such as disruption of prostaglandin synthesis and even local irritation.^44,45^

Although air pollution is a major health hazard in many developing countries including China, our findings may suggest a novel risk factor for gastric ulcer. Further studies are needed to examine the role of PM_2.5_ on peptic ulcer development. In keeping with our current observation, we have previously shown that about 4% of patients with bleeding PUD were negative for *H pylori* and NSAIDs in Hong Kong.^13^ There has also been a marked increase in these “idiopathic ulcers” in the past years, with a rise from 4.2% in 1997 to 1998 to 18.8% in 2000.^46^ Consistent findings were reported across countries in Asia with the proportion of patients with idiopathic ulcers ranging from 10% to 30%.^14^ These studies point to a potential unidentified cause of idiopathic ulcers, which may be partly attributable to PM_2.5_ exposure.

We merged several large databases to derive the estimation of PM_2.5_ pollutant levels in this study. We estimated exposure in individual residential locations using both satellite information and information from the addresses including vertical height (estimated from floor level) of the subjects. In Hong Kong, it has been demonstrated that the concentration of PM is inversely related to the height above ground in an exponential decay pattern (eAppendix). By taking into account the spatial variation of exposure, the measurement can minimize the underestimation of adverse health effects.^8,47^ A thorough examination of the health effects of PM exposure by this spatial model has confirmed the presence of commonly detectable health effects of PM including hospitalization for cardiovascular and respiratory diseases, and the lack of associations between PM exposure and diseases previously shown to be unrelated to air pollution. All of these collectively provide support for the validity of our new findings of the associations between PUD and long-term PM_2.5_ exposure.

This study has some limitations. This study only included elderly subjects who were previously enrolled in the Elderly Health Service. However, most of the hospitalizations related to PUD in Hong Kong are attributed to elderly patients. The presence of cardiovascular disease or stroke would increase the consumption of aspirin which increase the risk of ulcer related hospitalization. To compensate for this, we have adjusted for baseline medical illnesses including cardiorespiratory diseases and diabetes, or hospitalization for ischemic heart diseases, stroke, COPD, and type 2 diabetes that would increase the use of aspirin in different sensitivity analyses. Moreover, we did not have any information on the *H pylori* status of the subjects including those individuals who were hospitalized for PUD since this information was not available in the central hospital database. Also, we did not have access to individual endoscopy findings. Although we have demonstrated a significant association between PUD hospitalizations and PM_2.5_ exposure, there was no correlation between PUD bleeding and PM_2.5_ exposure. The exact reason for this finding remains uncertain. Although bleeding is only one of the complications related to PUD, not all hospitalization related to PUD are due to bleeding. Intuitively, it may be necessary to have a larger sample size to demonstrate the potential effect of PM_2.5_ on ulcer bleeding. Also, we did not consider the effects of other pollutants such as sulfur dioxides, nitrogen oxides, or carbon monoxide since we utilized a unique spatial method in measuring PM exposure, which could not be extended to other pollutants. Personal exposure measurement based on personal monitor is needed instead of one based on monitoring stations. We were also not able to adjust for other environmental factors including noise and meteorological conditions which vary among geographical areas.

We have demonstrated for the 1st time an association between long-term PM_2.5_ exposure and hospitalization for PUD in elderly people. Further studies in different patient cohorts and regions are needed to clarify the potential role of PM_2.5_ on the development of PUD.

## Supplementary Material

Supplemental Digital Content
