# Supplementary material for: STROBE-Long-Term Exposure to Ambient Fine Particulate Air Pollution and Hospitalization Due to Peptic Ulcers
Source: Medicine (Baltimore). 2016 May 6;95(18):e3543. doi: 10.1097/MD.0000000000003543 (PMC4863781; doi:10.1097/MD.0000000000003543)
Supplement: Supplemental Digital Content [file medi-95-e3543-s001.docx]

**eAppendix: Estimation of PM_2.5_ exposure at location of residence including height from ground level:**

(2-dimensional)

1. We used NASA satellite monitored Aerosol Optical Depth (AOD) data. AOD indicates transparency of electromagnetic radiation in the troposphere andis highly correlated with PM concentration(http://neo.sci.gsfc.nasa.gov/view.php?datasetId=MODAL2_M_AER_OD). However the resolution of AOD spatial distribution in 10x10 km isnot high enough to estimate spatial distribution of PM exposure.
2. The Hong Kong University of Science and Technology (UST) has established the Light Detection and Ranging(Lidar) instrument to measure surface extinction coefficient (SEC) which indicates the transparency of electromagnetic radiation within 1 km of the ground levelover non-reflective surfaceswith adjustment for atmospheric conditions. The resolution is improved to 1x1 km which is high enough for estimation of person exposure to PM [1].
3. The Environmental Protection Department (EPD) measured PM_2.5_in 3-4 stations from 1998 to 2001 (plots of PM_2.5_ in the EPD station). We regressed SEC obtained during dry conditions onPM_2.5_measured in EPD stations to derive the relationship between them. As SEC is in 1x1 km resolution, we can estimate the spatial distribution of PM_2.5_ at 1x1 km resolution [2].
4. Wehave assessed the validity of the method by building the regression model from 3 stations and predict the concentrations at the 4th station from year 2000 to 2011. We found that the predicted annual PM_2.5_was different from the data measured in EPD stations by around 9-12% (defined by the mean absolute difference/mean i.e. 3.7/32.0; 3.5/37.2; 4.3/36.1 and 4.5/38.8, respectively for each of the 4 stations) and the Root Mean Squared Error of 4.4–5.4 in the prediction with mean of 32.0–38.8. These validity measures were comparable to those obtained in US studies[3,4].

(3-dimensional including height from ground level)

1. In Hong Kong there were altogether 15 stations measuring PM_2.5_ in recent years. Their height (H) from ground level range from 3 to 27.5 m. We found that the estimated annual concentrations of PM_2.5_ were related to the height in an exponent fashion as shown in Figure A below.

Figure A:


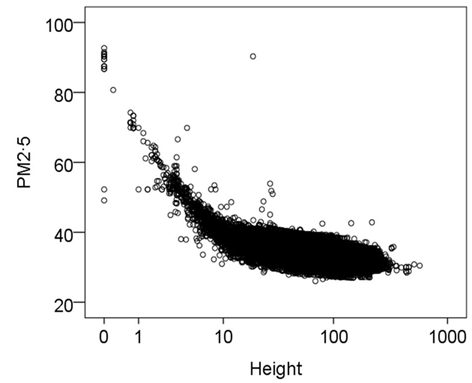


1. We then added the term “1/H” in the regression equation mentioned in No. 4 above and updated the regression equation for estimation of PM_2.5_ exposure of participants at their residential location including height.
2. We assessed the validity of this regression equation by comparison with the PM_10_concentrations with those obtained at a sample of locations and with a deterministic model which were studied in an independent MPhil study [5]. The results(in Table A below) showed that our estimated PM_10_ was comparable to that of the PM_10_ modeled from AirGIS/OSPM.As we did not have the data for PM_2.5_ modeled from AirGIS/OSPM model, we could not do similar comparison for PM_2.5_. However, we expect that the results would be similar for PM_2.5_.

Table A: Validating estimated values (μg/m^3^) with AirGIS/OSPM

|  | **AirGIS/OSPM** | | | **Exposure model** | | |
| --- | --- | --- | --- | --- | --- | --- |
|  | **Street** | | | **Street** | | |
| **Height** | **1** | **2** | **3** | **1** | **2** | **3** |
| **3m** | 60.3 | 59.5 | 59.0 | 61.4 | 54.5 | 56.7 |
| **15m** | 56.1 | 55.3 | 53.5 | 55.7 | 53.0 | 54.4 |
| **30m** | 54.3 | 53.8 | 53.5 | 53.0 | 51.9 | 53.0 |
| **45m** | 53.0 | 52.4 | 53.5 | 51.8 | 51.2 | 52.1 |
| **60m** | 52.8 | 52.3 | 53.5 | 51.0 | 50.8 | 51.6 |

Footnotes:

1. Hong Kong University of Science and Technology.Satellite Informatics System for Surface Particulate Matter Distribution.Available:<http://envf.ust.hk/itf-si/>

2. Wong CM, Lai HK, Tsang H, Thach TQ, Thomas GN, Lam KB, et al. Satellite-Based Estimates of Long-Term Exposure to Fine Particles and Association with Mortality in Elderly Hong Kong Residents. Environ Health Perspect. 2015. In press. Supplementary Figure S1 (<http://ehp.niehs.nih.gov/wp-content/uploads/advpub/2015/4/ehp.1408264.s001.acco.pdf>)

3. Krewski D, Jerrett M, Burnett RT, Ma R, Hughes E, Shi Y, et al. Extended follow-up and spatial analysis of the American Cancer Society study linking particulate air pollution and mortality. Res Rep Health Eff Inst. 2009;140: 5-114; discussion 115-36.

4. Ross Z, English PB, Scalf R, Gunier R, Smorodinsky S, Wall S, et al. Nitrogen dioxide prediction in Southern California using land use regression modeling: potential for environmental health analyses. J Expo Sci Environ Epidemiol. 2006;16:106-14.

5. Chapmen PS. The outdoor horizontal and vertical variations of respirable suspended particulate concentrations within a densely urban environment in Hong Kong - application of a box and plume dispersion model (airGIS/OSPM).MPhil. Thesis. The University of Hong Kong. 2012. Available:<http://sunzi.lib.hku.hk/ER/detail/space/4786984>

**eAppendix Figure 1: PM_2.5_ among the EPD monitoring stations in 1999-2011**


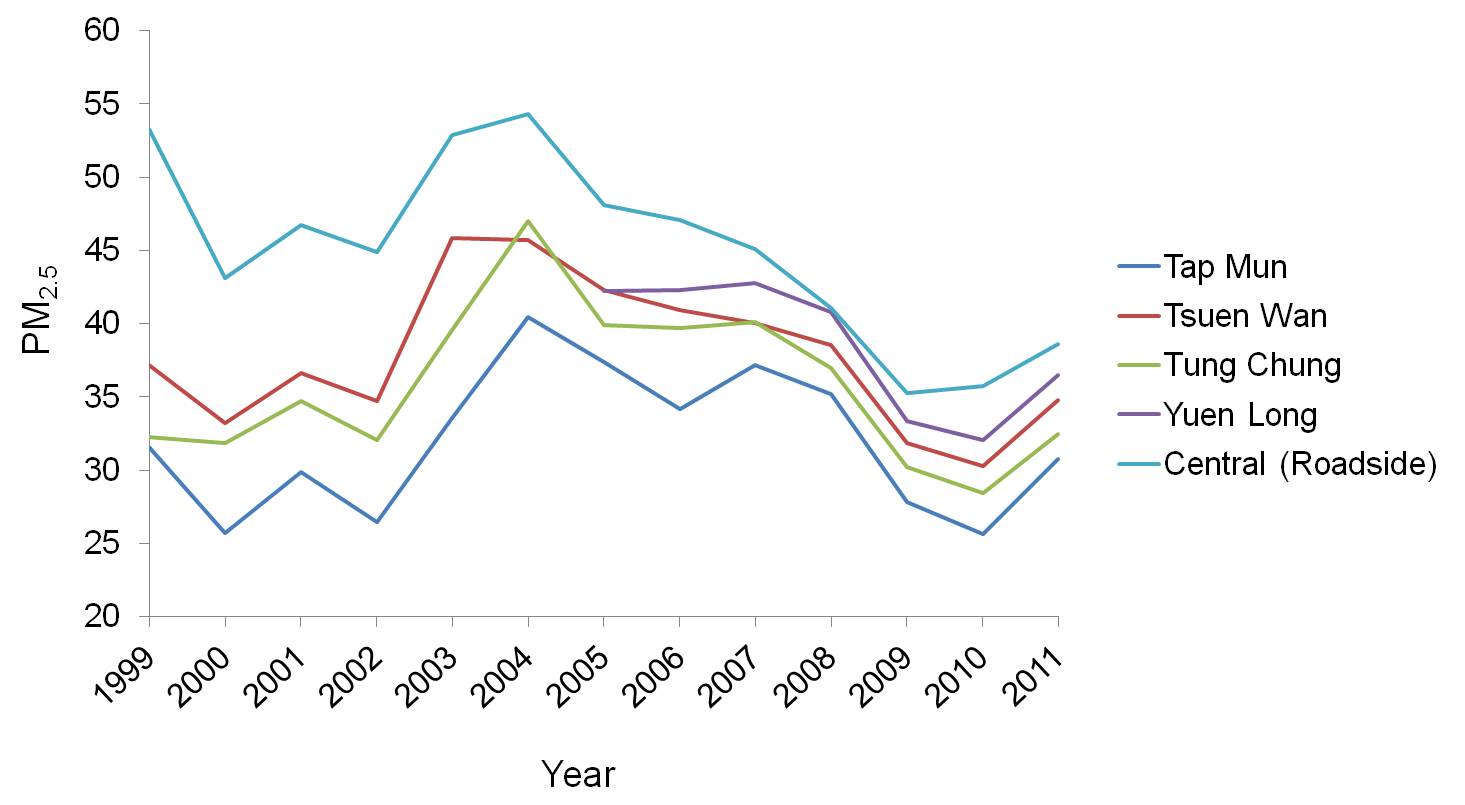


**eAppendix Figure 2. Monthly hospitalizations for PUD, 2001 - 2011.**


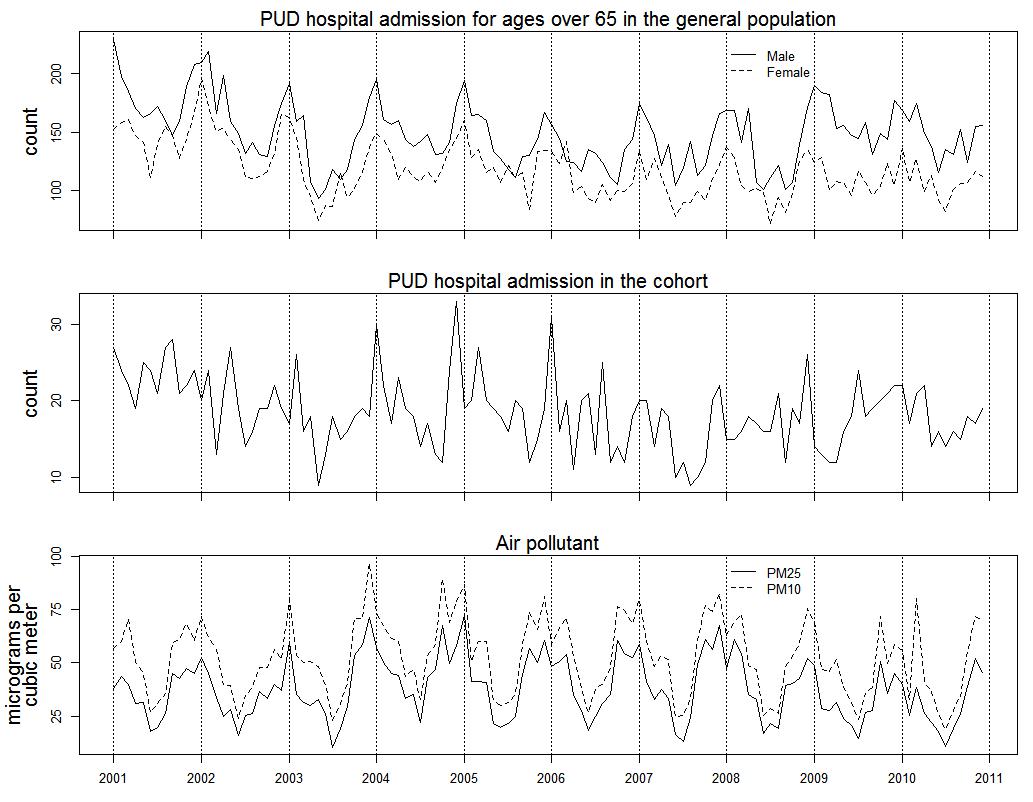


*Vertical lines indicate January each year.

**eAppendix Table 1**. Descriptive statistics of the participants by gender.

| **Variables** | **Male (N=20,674)** | **Female (N=39,599)** |
| --- | --- | --- |
| PM_2.5_ concentration (μg/m^3^) | 33.7±3.0 | 33.7±3.2 |
| Subject (%) | 34.3% | 65.7% |
| Floor number (mean±SD) | 11.3±9.0 | 11.3±9.2 |
| Age (mean year±SD) | 70.5±5.5 | 70.6±5.9 |
| BMI quartiles: Q2-Q3: 21.6 - 26.3 | 53.4 | 49.2 |
| Q1: <21.6 | 24.4 | 22.4 |
| Q4: >26.3 | 22.2 | 28.4 |
| Smoking: Never | 38.1 | 88.2 |
| Quitted | 41.4 | 7.8 |
| Current | 20.5 | 4.0 |
| Exercise days/week (mean±SD) | 5.4±2.7 | 5.6±2.6 |
| Education: Secondary or above | 30.6 | 10.2 |
| Primary | 51.3 | 29.3 |
| Below primary | 18.1 | 60.5 |
| Expense/month in US$ <128 | 11.5 | 16.1 |
| 128-384 | 68.0 | 65.7 |
| ≥385 | 20.5 | 18.2 |
| TPU level: Age≥65 (mean% ±SD) | 12.1±4.2 | 12.1±4.2 |
| TPU level: Tertiary education (mean% ±SD) | 13.2±8.0 | 12.9±8.0 |
| TPU level: Income≥US$1,923/m (mean% ±SD) | 59.7±11.6 | 59.4±11.6 |
| District level: ETS (mean% of smokers ±SD) | 11.5±0.4 | 11.6±0.4 |
| Grid level: Radon in kBqm^-3^0-40 | 16.0 | 16.1 |
| 41-100 | 66.0 | 65.7 |
| ≥101 | 18.0 | 18.2 |

**Source:**

Wong CM, Tsang H, Lai HK, et al. Cancer Mortality Risks from Long-Term Exposure to Ambient Fine Particle. *Cancer Epidemiol Biomarkers Prev*. In press. Accepted on 24 February 2016.

| **eAppendix Table 2: Summary statistics of air pollutant and meteorological measurements in Hong Kong (1998-2007) (N=3,652 days)** | | | | | | | | | |
| --- | --- | --- | --- | --- | --- | --- | --- | --- | --- |
|  | | | Mean | SD | Percentiles | | | | |
|  | | |  |  | Min | 25th | 50th | 75th | Max |
| Air pollutant () 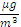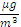 | | |  |  |  |  |  |  |  |
|  | PM_10_ | | 54.078 | 29.035 | 4.000 | 31.458 | 48.083 | 71.125 | 253.955 |
|  | Al | | 0.255 | 0.259 | 0.004 | 0.089 | 0.180 | 0.330 | 2.900 |
|  | As | | 0.005 | 0.006 | 2.6x10^-4^ | 4.9 x10^-4^ | 0.003 | 0.007 | 0.089 |
|  | Ca | | 0.770 | 0.650 | 0.012 | 0.360 | 0.590 | 0.960 | 8.300 |
|  | Cd | | 0.002 | 0.003 | 6.0 x10^-5^ | 2.4 x10^-4^ | 0.001 | 0.003 | 0.080 |
|  | Cl^-^ | | 0.925 | 1.115 | 0.002 | 0.215 | 0.530 | 1.200 | 12.000 |
|  | EC | | 4.113 | 2.010 | 0.240 | 2.800 | 3.900 | 5.100 | 24.000 |
|  | Fe | | 0.531 | 0.401 | 0.010 | 0.260 | 0.430 | 0.680 | 4.000 |
|  | K^+^ | | 0.611 | 0.580 | 0.110 | 0.180 | 0.420 | 0.870 | 6.000 |
|  | Mg | | 0.259 | 0.178 | 0.004 | 0.140 | 0.220 | 0.320 | 2.700 |
|  | Mn | | 0.019 | 0.017 | 0.002 | 0.005 | 0.014 | 0.026 | 0.260 |
|  | Na^+^ | | 1.487 | 0.998 | 0.011 | 0.730 | 1.300 | 2.000 | 9.000 |
|  | Ammonium | | 4.624 | 4.080 | 0.022 | 1.424 | 3.839 | 6.667 | 38.388 |
|  | Ni | | 0.005 | 0.006 | 3.4 x10^-4^ | 0.002 | 0.004 | 0.007 | 0.140 |
|  | Nitrate | | 15.089 | 13.751 | 0.255 | 6.080 | 10.591 | 19.612 | 178.471 |
|  | OC | | 8.620 | 5.739 | 0.300 | 4.400 | 7.300 | 11.000 | 50.000 |
|  | Pb | | 0.064 | 0.075 | 0.001 | 0.010 | 0.042 | 0.095 | 1.800 |
|  | Sulfate | | 10.541 | 6.898 | 0.610 | 5.200 | 9.400 | 14.000 | 63.000 |
|  | V | | 0.011 | 0.012 | 4.8 x10^-4^ | 0.005 | 0.005 | 0.014 | 0.130 |
|  | |  |  |  |  |  |  |  |  |

**Source:**

1. Air Quality In Hong Kong 2010. Air Science Group. Hong Kong Environmental Protection Department. Available at: <http://www.aqhi.gov.hk/api_history/english/report/files/AQR2010e_final.pdf>

2. Yuan ZB, Lau AKH, Zhang HY, Yu JZ, Louie PKK, Fung JCH. Identification and spatiotemporal variations of dominant PM10 sources over Hong Kong. *Atmospheric Environment.* 2006;40:1803–15.
